# Updated programmatic learning outcomes for the training of family physicians in South Africa

**DOI:** 10.4102/safp.v63i1.5342

**Published:** 2021-09-06

**Authors:** Robert Mash, Hannes Steinberg, Mergan Naidoo

**Affiliations:** 1 Division of Family Medicine and Primary Care, Stellenbosch University, Cape Town, South Africa; 2 South African Academy of Family Physicians, Cape Town, South Africa; 3 Department of Family Medicine, University of the Free State, Bloemfontein, South Africa; 4 Department of Family Medicine, University of KwaZulu-Natal, Durban, South Africa; 5 South African College of Family Physicians, Cape Town, South Africa

**Keywords:** family physicians, health professions education, learning outcomes, education, curriculum, competency-based education

## Abstract

The training of medical specialists should constantly be re-aligned to the needs of the population and the health system. The national Education and Training Committee of the South African Academy of Family Physicians reached consensus on the updated programmatic learning outcomes for the training of specialist family physicians in South Africa. Learning outcomes were first developed to guide training programmes when the speciality was recognised in 2007. Fifteen years later, it was time to revisit and revise these learning outcomes. Learning outcomes define what family physicians are able to do at the end of 4 years of postgraduate training. This revision presents five unit standards and 83 programmatic exit-level learning outcomes.

## Introduction

The training of medical specialists should constantly be re-aligned to the needs of the population and the health system.^1^ In 2021, the discipline of Family Medicine in South Africa presents an updated set of programmatic learning outcomes for the training of specialist family physicians, which defined what family physicians should be able to do at the end of their 4 years of postgraduate training.

These new outcomes replace the previous ones that were published in 2012.^2^ The original outcomes were developed simultaneously with the recognition of Family Medicine as a new specialty in South Africa in 2007. Over the last 10-years, the discipline has observed the actual competencies expected of family physicians as they engage with patients and the health system, mostly at a primary health care and district hospital level.^3^ The health system has evolved through reengineering and strengthening of primary health care as well as through the piloting of national health insurance as a vehicle to enable universal health coverage.^4^ The landscape of the burden of disease has also shifted with, for example, the human immunodeficiency virus (HIV) epidemic being more controlled through antiretroviral medication and non-communicable diseases, such as diabetes mellitus, becoming more prominent.^5^

During the last 10-years, the university training programmes have coordinated their activities through the South African Academy of Family Physician’s national Education and Training Committee (ETC). At the same time, the College of Family Physicians, within the Colleges of Medicine of South Africa, was mandated to run the national licensing examination. Over this period, a number of revisions were already approved, such as new outcomes for training in leadership and clinical governance,^6^ as well as a new list of clinical skills.^7^

The national outcomes should be aligned with the curriculum content, educational approach and forms of assessments within each training programme. These new outcomes will, therefore, have implications for each university. Workplace-based training and assessment is an essential component of all programmes; and a standardised national portfolio of learning has been introduced across all programmes.^8^ This in turn is one of the entry requirements for the licensing examination.

## Process of revision

The previous unit standards and outcomes were included in an electronic questionnaire that went to all training programmes in the country. Each training programme discussed the outcomes internally and gave feedback in the questionnaire on the structure of the unit standards and whether to keep, discard or re-phrase each outcome. In addition, every training programme had the opportunity to suggest new learning outcomes. The collated results were presented to the ETC, and all changes and comments were discussed. From the results of the survey, it was recognised that unit standard three, on community-orientated primary care,^9^ needed more extensive revision and careful thought. A sub-group was tasked with re-writing these outcomes completely and presenting the results to the ETC. At the same time, a sub-group, assessing the curriculum content for unit standard four, also made recommendations on revising the learning outcomes. The final set of outcomes were discussed again by the ETC, and a consensus was reached across all training programmes.

## Unit standards and learning outcomes

The five unit standards were retained, but the definitions were revised as shown in Table 1. The learning outcomes for each unit standard are presented in Table 2.

**TABLE 1:** Definitions of unit standards and capabilities required.

Unit standard definitions	Unit standard capabilities
Unit standard 1: Effectively manage themselves, their team and their practice, in any sector, with visionary leadership and self-awareness in order to ensure the provision of high-quality, evidence-based care.	A person who has achieved this standard is capable of effectively managing themselves, their team and their practice, regardless of the sector, shows self-awareness in their personal and professional approach and provides high-quality care based on current evidence
Unit standard 2: Evaluate and manage patients with both undifferentiated and more specific problems in a holistic, cost-effective manner.	A person who has achieved this standard is capable of evaluating and managing patients, with both undifferentiated and more specific problems, holistically and cost-effectively.
Unit standard 3: Improve the health and quality of life of the community.	A person who has achieved this standard is capable of leading and implementing integrated and comprehensive community-orientated primary care.
Unit standard 4: Facilitate the learning of others regarding the discipline of family medicine, primary healthcare, and other health-related matters.	A person who has achieved this standard is capable of educating, teaching, mentoring or supervising others regarding the discipline of family medicine, primary healthcare, and other health-related matters. For example, this may involve the supervision of clinical associates, interns or registrars, teaching of medical students or mentoring of clinical nurse practitioners and junior medical officers. The capability also extends to interaction with community groups and patients.
Unit standard 5: Conduct all aspects of healthcare in an ethical, legal and professional manner.	A person who has achieved this standard is capable of conducting all aspects of healthcare in an ethical, legal and professional manner.

**TABLE 2:** Programmatic learning outcomes.

Unit Standard	Description
Unit standard 1	Effectively manage themselves, their team and their practice, in any sector, with visionary leadership and self-awareness in order to ensure the provision of high-quality, evidence-based care.
1.1 Developing self optimally as a leader:	1.1.1 Demonstrating self-awareness and reflection in terms of one’s personality, personal values, preferred learning and leadership styles, and learning and development needs 1.1.2 Demonstrating effective methods of self-management and self-care 1.1.3 Demonstrating willingness to seek help, when necessary 1.1.4 Demonstrating an ability to implement and monitor strategies for self-growth and personal development
1.2 Offer leadership within the healthcare team and district health system by:	1.2.1 Communicating and collaborating effectively 1.2.2 Demonstrating an ability to build capability, mentor or coach members of the healthcare team 1.2.3 Demonstrating an ability to engage and influence others through advocacy, group facilitation, presentations, critical thinking, or behaviour change counselling 1.2.4 Working effectively as a member of the sub/district healthcare team
1.3 Describe and contribute to the functioning of the district healthcare system	1.3.1 Demonstrating an understanding of the principles of the district health system in the context of existing and developing national legislation and policy 1.3.2 Demonstrating an ability to contribute to the management of a facility, sub-district, or district
1.4 Lead clinical governance activities	1.4.1 Demonstrating the ability to lead a quality improvement cycle in practice 1.4.2 Facilitating reflection on health information (e.g. monitoring and evaluation, national core standards) in order to improve quality of clinical care (e.g. rational prescribing and use of investigations) in the sub-district/district 1.4.3 Facilitating risk management processes and improving patient safety (e.g. conduct morbidity and mortality meetings, assess competence of new clinical staff, perform root cause analysis, manage patient complaints) in the sub-district/district 1.4.4 Facilitating the implementation of clinical guidelines in the sub-district/district 1.4.5 Critically reviewing new evidence (e.g. research) and applying the evidence in practice 1.4.6 Contributing to the development or revision of guidelines by generating new evidence (e.g. perform research) or representing the viewpoint of the district health services in the process
1.5 Understand and influence corporate governance	1.5.1 Understand the principles of human resource management (e.g. labour relations, recruitment, disciplinary procedures, grievances) 1.5.2 Demonstrate the ability to complete performance appraisals of staff 1.5.3 Understand the principles of financial management (e.g. budgets, health economics, financial planning) 1.5.4 Understand the principles of procurement and infrastructure (e.g. supply chain, equipment, buildings) 1.5.5 Understand the principles of health information and record-keeping systems 1.5.6 Understand the principles of rational planning of health services 1.5.7 Be able to communicate effectively with those responsible for corporate governance
Unit standard 2	Evaluate and manage patients with both undifferentiated and more specific problems in a holistic, cost-effective manner.
2.1 Evaluate a patient holistically:	2.1.1 Taking a relevant history in a patient-centred manner, including exploration of the patient’s illness experiences and context. 2.1.2 Performing a relevant and accurate examination. 2.1.3 Deciding on or performing appropriate special investigations where indicated, based on current evidence and balancing risks, benefits and costs. 2.1.4 Formulating a holistic assessment of the patient’s problems, taking into account the biological, psychological, spiritual, social and contextual issues 2.1.5 Demonstrating sound clinical reasoning at every point in the consultation.
2.2 Formulate and execute, in consultation with the patient, a mutually acceptable, cost-effective management plan, evaluating and adjusting elements of the plan as necessary by:	2.2.1 Communicating effectively with patients to inform them of the diagnosis or assessment and to seek consensus on a management plan 2.2.2 Establishing priorities for management, based on the patient’s perspective, biological and socio-economic preconditions, medical urgency and context. 2.2.3 Formulating a cost-effective management plan and appropriate safety netting. 2.2.4 Formulating a management plan for patients with family-orientated or other social problems, making appropriate use of family and other social and community support, and resources. 2.2.5 Applying technology cost-effectively and in a manner that balances the needs of the individual patient and the greater good of the community. 2.2.6 Incorporating disease prevention and health promotion. 2.2.7 Performing effectively and safely the procedural and surgical skills necessary to function as a generalist. 2.2.8 Effectively managing concurrent, multiple and complex clinical issues, both acute and chronic, often in a context of uncertainty. 2.2.9 Demonstrating a patient-centred approach to management using collaborative decision-making. 2.2.10 Including the family in the management and care of patients, whenever appropriate. 2.2.11 Counselling patients, for example, with regard to bad news, psychosocial issues, trauma, behaviour change and difficult decisions. 2.2.12 Recognising and managing the importance of relationships that affect health, using appropriate tools (e.g. genograms and ecomaps) to identify potential problems and solutions. 2.2.13 Demonstrating the ability to work in collaborative multidisciplinary teams 2.2.14 Referring patients, where necessary, to the appropriate level of care or expertise 2.2.15 Coordinating the care of patients with other professionals or health workers 2.2.16 Demonstrating appropriate recordkeeping
2.3 Provide comprehensive, continuing care throughout the lifecycle, incorporating preventative, diagnostic, therapeutic, palliative and rehabilitative interventions, by:	2.3.1 Demonstrating a commitment to building continuity of care and ongoing relationships with patients, as well as an understanding of the chronic care model. 2.3.2 Demonstrating an ability to provide preventive care, using primary, secondary and tertiary prevention as appropriate, and to promote wellness. 2.3.3 Demonstrating the ability to make a functional assessment of a patient with impairment or disability and enable his or her rehabilitation. 2.3.4 Demonstrating the ability to provide holistic palliative care. 2.3.5 Demonstrating an understanding of the emotional and physical aspects of pregnancy, birth, childhood, adolescence, young adulthood, adulthood and aging.
Unit standard 3	Improve the health and quality of life of the community.
3.1. Lead and support the implementation of community-orientated primary care (COPC)	3.1.1 Explain the principles of COPC to other healthcare workers and managers 3.1.2 Collaborate with the local management team to plan and implement COPC 3.1.3 Assist with the interpretation of data on the community’s health assets and health needs 3.1.4 Assist with the prioritisation of health issues in the community 3.1.5 Assist with the planning of responses or interventions to address these health issues in the community 3.1.6 Participate in the implementation of these responses or interventions in the community 3.1.7 Assist with the evaluation of these responses or interventions
3.2 Provide support to the primary health care (PHC) teams in your community	3.2.1 Act as a clinical consultant to the whole PHC team, including issues arising from CHW teams in the community. 3.2.2 Be able to conduct home visits in support of the PHC team when necessary. 3.2.3 Lead clinical governance activities (see unit standard 1#) for the whole PHC team, including the CHW teams in the community.
3.3 Link the PHC teams to the rest of the health system, other sectors and community	3.3.1 Ensure coordination of care within and between PHC teams. 3.3.2 Ensure coordination of care between PHC teams and the district hospital and higher levels of care with clear referral pathways. 3.3.3 Build relationships between the PHC teams and other sectors, particularly social services, in order to enable inter-sectoral engagement in health issues and improve safety for the PHC teams. 3.3.4 Build relationships between the PHC teams and community structures or forums in order to enable community engagement and participation in health issues and improve safety for the PHC teams.
Unit standard 4	Facilitate the learning of others regarding the discipline of family medicine, PHC, and other health-related matters.
4.1 Demonstrate the role of the family physician as a teacher:	4.1.1 Contribute to the development of an organisational learning environment 4.1.2 Assessing the learning needs of others and planning educational activities. 4.1.3 Conducting effective learning conversations in the workplace 4.1.4 Using appropriate and up-to-date educational technology effectively. 4.1.5 Deliver an effective educational presentation. 4.1.6 Facilitating small group learning. 4.1.7 Eliciting course evaluation and feedback from participants or students. 4.1.8 Applying the principles of assessment of learning. 4.1.9 Conducting an evidence-based approach to teaching. 4.1.10 Managing the learner in difficulty.
Unit standard 5	Conduct all aspects of healthcare in an ethical, legal and professional manner.
5.1 Demonstrate an awareness of the legal and ethical responsibilities in the provision of care to individuals and populations by:	5.1.1 Identifying and defining an ethical dilemma using ethical concepts. 5.1.2 Applying a problem-solving approach in which the law, ethical principles and theories, medical information, societal and institutional norms, and personal value system are reflected. 5.1.3 Formulating possible solutions to the ethical dilemma. 5.1.4 Implementing these solutions in order to provide healthcare in an ethical, compassionate and responsible manner that reflects respect for the human rights of patients and colleagues. 5.1.5 Demonstrating adherence to Health Professions Council of South Africa ethical guidelines 5.1.6 Apply relevant law to clinical practice 5.1.7 Demonstrate the ability to effectively manage patient complaints and advise on medico-legal risks and media enquiries.
5.2 Demonstrate professional values in relationship to society, interpersonal relationships and personal behaviour by:	5.2.1 Demonstrating professional values in relationship to society, for example, striving for equity in healthcare delivery, striving for quality in healthcare delivery and defending the human rights of patients and colleagues. 5.2.2 Demonstrating professional values in interpersonal relationships, for example, dealing courteously with patients, colleagues and the public, and having regard for cultural issues and individual dignity. 5.2.3 Demonstrating professional values in personal behaviour, for example, delivering healthcare of a consistent high standard irrespective of his or her own perceptions or prejudices and the background, with respect to gender, ethnicity, religion or sexual orientation, of his or her patient.

CHW, community health worker.

## Conclusion

This open forum article, presents the updated programmatic learning outcomes for training family physicians in South Africa as agreed by the discipline in 2021. Departments of Family Medicine at all universities that train family physicians will need to ensure that their programmes are re-aligned with these new outcomes. The College of Family Physicians will also need to ensure that in future the blueprinting of their national licencing examination is aligned with the new outcomes. Plans are underway to align the learning outcomes with a set of entrustable professional activities and observable practice activities. The outcomes will be revised in 5 years’ time.
